# Structural and Rheological Characterization of Vegetable Crispbread Enriched with Legume Purée

**DOI:** 10.3390/molecules29081880

**Published:** 2024-04-20

**Authors:** Karolina Szulc, Sabina Galus

**Affiliations:** Department of Food Engineering and Process Management, Institute of Food Sciences, Warsaw University of Life Sciences—SGGW, Nowoursynowska Str. 159c, 02-776 Warsaw, Poland; karolina_szulc1@sggw.edu.pl

**Keywords:** vegetable crispbread, legumes, purée, citrus pectin, physical properties

## Abstract

Crispbread is gaining popularity as a healthy snack or bread substitute. This is a lightweight dry type of flat food that stays fresh for a very long time due to its lack of water and usually contains different types of grain flour, including gluten-containing wheat or rye flour. The incorporation of legume purée into crispbread represents an innovative approach to enhancing the nutritional profile and taste of the product. The rheological properties of various legume purées (chickpea, white bean, black bean, and red bean) mixed with citrus pectin were examined, revealing significant differences in fluid behavior and viscosity. Crispbread formulations were analyzed for water content and activity, color, structure, FT-IR spectra, water vapor adsorption isotherms, and sensory evaluation. The results showed the possibility of obtaining crispbread based on the purée of legumes and citrus pectin. Crispbread enriched with red bean purée exhibited low water activity (0.156) and water content (3.16%), along with a continuous porous structure, and received the highest sensory evaluation score among the products. These findings can be treated as a basis for the development of other innovative recipes and combinations using legumes.

## 1. Introduction

Legumes such as beans, peas, chickpeas, lupins, and lentils are good and sustainable sources of protein, dietary fiber, carbohydrates, B vitamins, minerals and antioxidants [1,2,3,4,5]. They have been used in enriched foods and have been recognized as a vital food resource for the human diet, reducing the risk of cardiovascular disease, cancer and diabetes [6].

Legume-based ingredients (flour, starch, protein and fiber) have great potential in food formulation to improve the nutritional quality of foods due to their high protein and fiber content, gluten-free status and low glycemic index, as well as functional properties [7,8]. For example, the functional properties of legume proteins include water binding capacity and solubility, emulsification, foaming and gelation [9,10,11,12,13,14]. These functional properties have allowed the use of proteins as components of flour and other whole legume products in beverages, soups, snacks, bread, dressings or meat products [3,15,16,17,18,19]. Incorporating legume-based ingredients into a variety of food products has the potential to expand the use of legumes beyond traditional uses and consumption patterns. The protein sources of legumes play a key role in the development of gluten-free bakery products. The high solubility of legume proteins in the aqueous phase is an important factor that positively influences the preservation of other functional properties in a gluten-free system [20]. Furthermore, the inclusion of legumes in bakery products is important, both for people with celiac disease and for people sensitive to gluten, as a strategy to increase nutritional value and expand dietary options [21,22].

Legumes can be used in various forms, such as aquafaba, extracts, flours, bran, textured proteins, and sprouts, to create a wide range of food applications. Recently, several studies have been carried out to improve the quality and nutritional value of foods by incorporating legume ingredients into these products, including bread, cakes, cookies and other baked or cooked products [22,23,24]. For example, López-Martínez et al. [25] used mixtures of common bean, broad bean, and textured soybean flour in different proportions to produce chips. The formulation most accepted by consumers was made of 65% broad bean flour and 35% soybean flour. Additionally, this sample had the highest protein and fiber content. The addition of broad beans to the formulations increased the digestibility of the product.

Beans and other legumes are very easy to purée. Their processing into puréed products creates opportunities for puréed beans to be incorporated into a variety of products such as baby foods, specialty foods for consumers with age-related chewing and swallowing disorders, and for other applications in conventional and functional foods. For example, by adding legume purée to bread recipes. In the study by Kasapila et al. [26], the standard recipe was modified and bread was developed with 20, 30 and 50% wheat flour replaced by bean purée. Participants rated the 20% bean purée bread as the most acceptable in terms of appearance, flavor, and taste. The enriched bread was slightly denser than traditional bread, based on 100% wheat flour, and the bean purée increased its nutritional value. Puréed legumes can also be used to replace fat in baked foods. In a study conducted on brownies, black bean purée effectively replaced fat with a sensory acceptability rating of 30% replacement. Furthermore, this study showed that replacing 90% of fat with black beans still produced an acceptable product [27]. The use of green pea purée as a fat replacement at 25% in chocolate bar cookies was considered optimal; the recipe turned out to be better in terms of sensory attributes compared to the control [28]. Butter bean purée was found to be effective in terms of an acceptable and likable replacement of fat in sponge cake up to the 50% level [29].

Crispbread is treated as a traditional substitute for bread or as a snack and is considered a functional food. The production of crispbread is based on the traditional method (baking) or extrusion. The crispbread market includes the following types of products: regular crispbread, light crispbread (extruded), rusks and rice bread [30]. Crispbread is a versatile food item that can be eaten in various ways. It can serve as a base for spreads, cheeses, or toppings, making it a popular choice for appetizers, snacks, and light meals. Crispbread can also be paired with soups, salads, or charcuterie boards to add texture and crunch. In addition, it is a convenient option to snack on the go or as a component of packed lunches. Crispbread made from legumes could be a healthier alternative to wheat flour baked goods, which are characterized by a high glycemic index and low protein content [20,31]. With the incorporation of citrus pectin, a soluble dietary fiber derived from citrus fruits, into vegetable crispbread formulations, the opportunity arises to not only improve its structural integrity, but also increase its health-promoting properties. Citrus pectin, derived from a variety of citrus fruits such as oranges, lemons, grapefruits, and limes, is a type of highly esterified pectin with a high content of ester groups. It can form gels and thicken liquids, making it widely used in the food industry as an ingredient in the production of jams, marmalades, jellies, sauces, and fruit drinks. Citrus pectin adds the right consistency, stability, and viscosity to these products. Furthermore, it can be obtained from waste after processing citrus fruits, helping to reduce waste and conserve natural resources [32].

So far, there have been no reports on the use of legume purée in the production of gluten-free crispbreads. Therefore, the objective of this study was to characterize (rheology, water content and activity, color, structure, water adsorption isotherms, FT-IR and sensory evaluation) vegetable crispbread based on legume purée.

## 2. Results and Discussion

### 2.1. Rheological Properties of Legume Solutions

**Figure 1:**
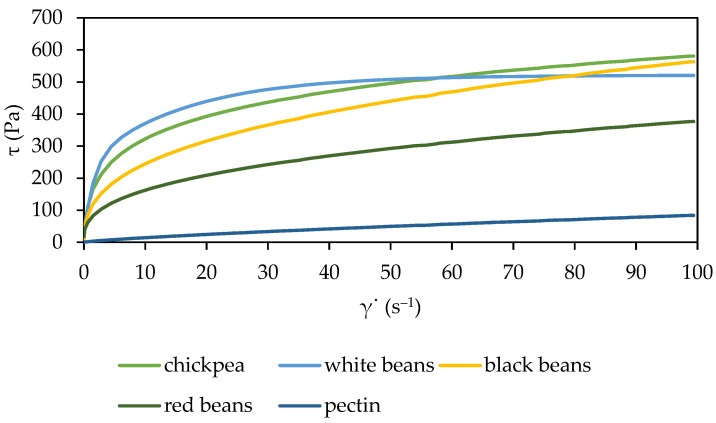
Flow curves of legume solutions.

The rheological properties of purées play an important role in determining their suitability for various food applications, such as bakery, sauces, soups, and beverages. Rheology refers to the study of the flow and deformation of materials under stress, and understanding the rheological behavior of the material helps optimize processing conditions and ensure desired product attributes [33,34]. Figure 1 shows the flow curves of solutions of high-methoxyl citrus pectin with legume purée (chickpea, white, black, and red beans). The addition of legume purée to the citrus pectin solution increased shear stress at the same shear rate. The flattest flow curve was observed for the pectin solution with red bean purée. The highest values of shear stress, along with an increase in shear rate, were reported for a solution of citrus pectin mixed with white bean purée at a shear rate of approximately 65 s^−1^; beyond this value, stabilization of the system was observed. In the case of the remaining solutions, there was a continuous increase in the shear stress value along with an increase in the shear rate. Citrus pectin solutions with different legume purées demonstrated nonlinear curves passing through the center of the coordinate system, which allowed them to be classified as shear-thinning liquids. This group of liquids also includes the citrus pectin [35]. In addition, the shear-thinning characteristics became more pronounced after adding the legume purée to the citrus pectin solution. According to Mohammed et al. [36], high methoxyl pectin solutions at a concentration of up to 1% have the characteristics of Newtonian liquids; however, with the change in pectin concentration (2%), the solution transforms into a shear-thinning fluid. Similar shear-thinning behavior has also been reported in previous studies on mango pulp [37], citrus peels [38], dragon fruit purées [39], and carrot purée with the addition of 0.8% highly esterified pectin [40] and bean flour dispersions [41].

**Figure 2:**
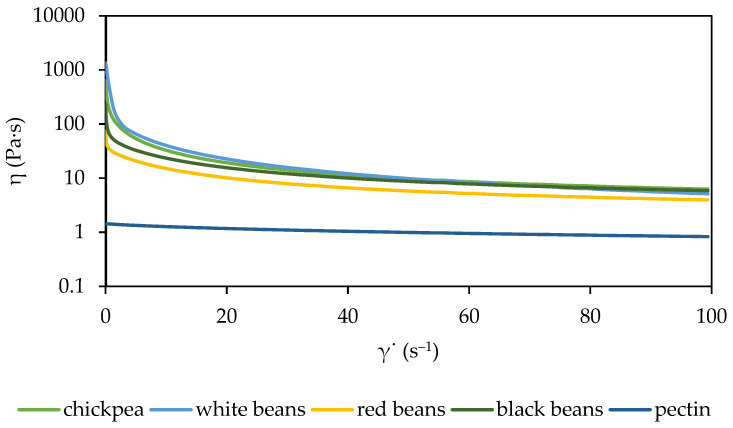
Viscosity curves of legume solutions.

The apparent viscosity of the solutions decreased with increasing shear rate (Figure 2). The addition of purée to the citrus pectin solution increased the viscosity, and its value depended on the type of legume. At low shear rates (up to 10 s^−1^), significant differences in viscosity between samples were observed, and at high shear rates, minor differences were detected. This was also confirmed by tests conducted on different pea purée samples [42]. Paste made from four types of legumes (soybean, red bean, kidney bean, and mung bean) was studied by Pang et al. [43] in terms of its rheological properties. The soybean paste showed lower viscosity and consistency coefficients than the other pastes. In turn, the red bean paste presented the highest viscosity. In the present study, citrus pectin solutions with white bean purée and chickpea purée were characterized by the highest viscosity (Table 1). The addition of white bean purée contributed to the highest increase in apparent viscosity (more than 100 times) compared to the pectin solution. The viscosity curves of pectin from apple pomace [44] presented a similar course to the viscosity curve of the citrus pectin solution presented in Figure 2. Viscosity differences may be due to the molecular weight of the pectin used [36]. The viscosity of all the samples was higher than the pea purée [42]. Janowicz et al. [45] examined the rheological properties of different hydrocolloid solutions (pork gelatin, soybean protein isolate, sodium alginate, and low and high methylated apple pectin) with pumpkin purée (40%). With an increase in the high concentration of methylated apple pectin (from 1 to 2%), an increase in the apparent viscosity of the solution was observed, ranging from 0.046 to 0.147 Pa·s. In this work, a much higher apparent viscosity of 1.02 Pa·s was obtained for a 5% citrus pectin solution (control sample), indicating different physicochemical properties of pectins, and is important in the area of applications of pectin in food [46].

In this work, the Oswald-de Waele model was used to describe the flow curves for the best fit (Table 1). The high value of the consistency coefficient (*k*) in the solution with the addition of white bean purée shows the lowest value of the flow index (*n*); similarly, the pectin solution with the addition of red beans showed the lowest value *k* and the highest value of *n*. The pectin solution without the addition of legumes shows the highest flow index and the lowest consistency coefficient; thus, it can be concluded that the addition of puréed legumes reduces the flow index and increases the consistency coefficient. *k* increases with the concentration of fluid food products, while *n* is close to 0.5 for pulpy products and close to 1 for clear juices, and it decreases slightly with concentration [47]. A relationship was observed between the increase in flow index and the decrease in apparent viscosity of the solution tested. At the highest apparent viscosity of the solution with the addition of white bean purée, the flow index has the lowest value. The solution with the addition of red bean purée had the highest flow index values among solutions with the addition of legume purée and the lowest apparent viscosity.

### 2.2. Water Content, Water Activity, and Color of Crispbread with Legume Purée

Depending on the formulation, the water content in the crispbread legumes ranges from 3.16 to 4.12% (Table 2). The results were consistent with other research related to legume chips (3.52–5.31 g/100 g) [25], crispbread with apple powder (3.87–4.25%) [48] and crispbread with pumpkin and carrot powder (3.96–5.04%) [49]. However, triticale crispbread had a higher water content (7.2–7.8%) [50] and the yellow split pea crackers showed the lowest values (1.28–3.16%) [51] than those presented in this study.

Samples were characterized by low water activity below 0.19 (Table 2). There were differences in the water activity of the product depending on the type of purée. The lowest water activity, 0.156, was found in crispbread based on white and red bean purée. Legume-based crispbreads were characterized by a lower water activity than crispbread analyzed by other authors [48]. This indicates that the composition of the raw material has a significant impact on the water activity of the final product. Low water activity helps preserve the product for a longer period and prevents the growth of microorganisms [52,53]. The maintenance of its microbiological stability depends on keeping the product free of excess. Therefore, crispbread with legume purée was considered shelf-stable.

For the consumer, the first moment of providing subjective information about the raw material, product, or process used to obtain it is visual assessment, including color assessment. The type of legume in the crispbread recipe influenced the changes in its color (Table 2). As expected, the sample with black bean purée had the lowest brightness and the sample with white bean purée had the highest brightness. Redness and yellowness dominate in snacks based on legume purée. The sample with red bean purée had the highest redness and the sample with chickpea purée had the highest yellowness. The legume-based crispbreads had more redness than extruded crispbread containing a mixture of flour and apple powder [48]. This can be explained by the presence of natural pigments in legumes [54,55,56]. However, color differences may also be due to the thermally induced protein-carbohydrate Maillard reaction [57,58]. Furthermore, in studies by other researchers, a decrease in brightness is observed with an increase in the proportion of legume flour [36,59].

### 2.3. Structure of Crispbread with Legumine Purée

**Figure 3:**
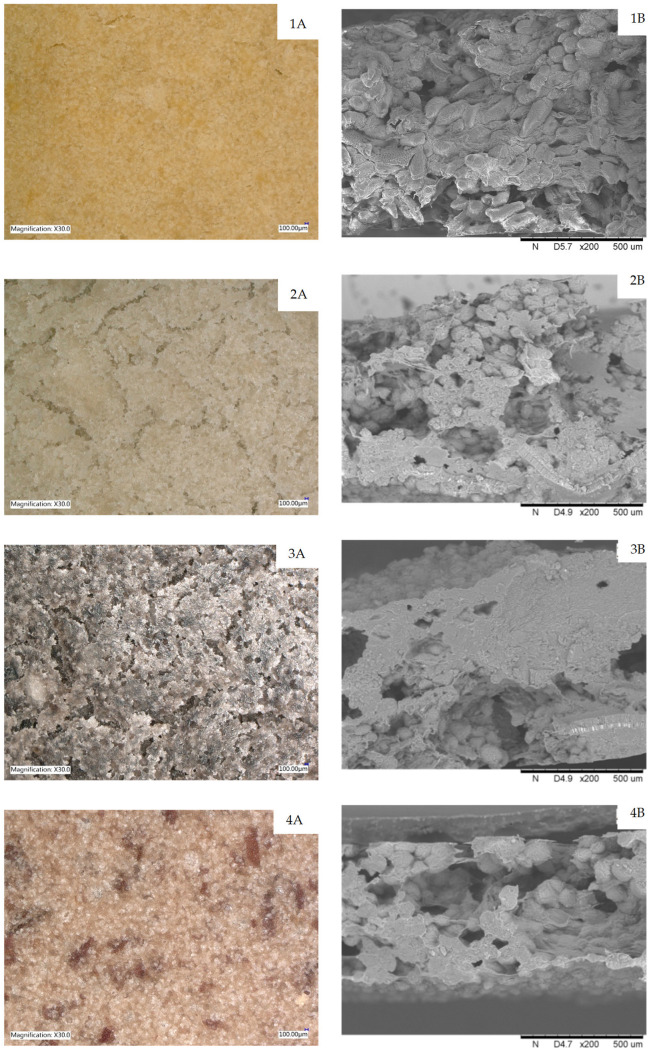
Structure of crispbread with legume purée: (**A**) surface; (**B**) cross-section, where 1—chickpea, 2—white beans, 3—black beans; 4—red beans.

Analyzing the structure of the crispbread presented in Figure 3, it was possible to notice differences in the surface morphology between the samples. Crispbread based on white bean and black bean purée was characterized by a non-uniform surface with numerous spaces (surface discontinuity). The chickpea purée sample and the red bean sample had a compact structure and a smooth surface. However, fragments of ground seed husks were visible in the crispbread with red bean purée. All cross-sectional samples showed free spaces along with visible starch granules from the legume. The amylose and amylopectin that structurally form the starch are deposited in discrete granules. These granules were of different sizes and shapes (disk-shaped and spherical shape). But the structure of the crispbread with black bean purée was very compact. In turn, in the case of the chickpea purée sample and the red bean sample, the most open and porous structure was observed.

The morphological properties of starch are also considered important factors in determining the quality of food products [60]. Wu et al. [61] observed that an increase in starch content causes a decrease in the specific volume of rice bread and an increase in its hardness. A low starch content and higher starch granule integrity produced better gluten-free rice bread. Starch consists of amylose and amylopectin and is deposited in the form of granules of various sizes and shapes. Starches from various sources are characterized by the variable chemical composition and structure of their ingredients. Smaller starch granules have a larger surface area, surface pores, and channels that increase water absorption. High hydration increases swelling, viscosity, and gelatinization ability of starch granules [62].

### 2.4. FT-IR Spectra of Crispbread with Legume Purée

**Figure 4:**
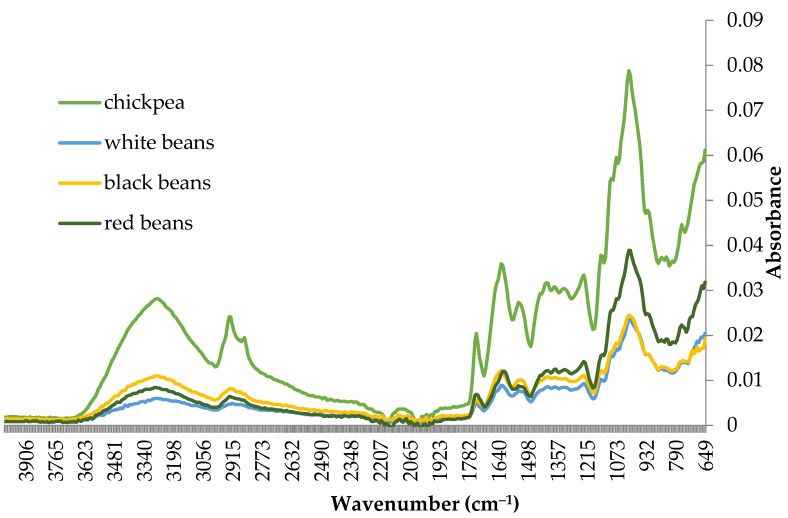
FT-IR spectra of crispbread with legume purée.

Fourier transform infrared spectroscopy (FT-IR) was used to study the structure of crispbread obtained from different legume purées (Figure 4). The addition of legume purée to citrus pectin reduced the intensity of the spectra. Furthermore, the differences in the intensity and position of the peaks in the crispbread spectra showed the effect of the type of legume purée. Crispbread with chickpea purée had the highest spectral intensity in the entire range compared to bean purée. For all samples, a broad peak of approximately 3000–3630 cm^−1^ was related to the stretching of the hydroxyl (–OH) groups of the galacturonic acid polymer [63], whereas the absorbance at approximately 2760–2980 cm^−1^ was attributed by distensions of the –CH_2_ and –CH_3_ groups and the stretching of –NH_3_ (amides B) [64]. This may be related to the presence of hydrophobic methylene groups in the proteins and carbohydrates of crispbread. The region 1480–1760 cm^−1^ deals with information about functional groups that occur in molecules. The distension C=O of the carbonyl groups (present in pectin) gave the absorption peak at wave 1715–1760 cm^−1^ [65]. Protein spectra, i.e., the Amide I (C–N) and Amide II (N–H) bands (around 1620 and 1530 cm^−1^, respectively), are the most intense. The other bands are of relatively low intensity and the proteins are practically transparent below 1400 cm^−1^ [66]. FT-IR spectra in the range from 840 to 1480 cm^−1^ create a ‘carbohydrate fingerprint’ region that allows the identification of the main chemical groups specific to individual polysaccharides. All samples had a characteristic band with a strong intensity at around 1000 cm^−1^ in the FT-IR spectra, which corresponded to starch [46,67,68,69]. The absorbance bands at 1022 and 1047 cm^−1^ are characteristic of amorphous and crystalline structures of starch, respectively. Hence, the ratio of 1047/1022 cm^−1^ was used to express the amount of ordered crystalline domains to amorphous domains in starches [70]. The ratio was lower for crispbread with red beans (0.830) than with white beans (0.835), chickpeas (0.841) and black beans (0.849). Ambigaipalan et al. [71] showed that the level of crystallinity of faba bean starch was lower than that of black bean or pinto bean starches. The addition of different purées can influence the crystalline and amorphous structure of starch through various mechanisms, such as mechanical pretreatment and interactions with the matrix. The degree of these changes depends on the type of starch, the composition of the purée, and the processing conditions [72].

### 2.5. The Water Vapor Adsorption Isotherms of Crispbread with Legume Purée

**Figure 5:**
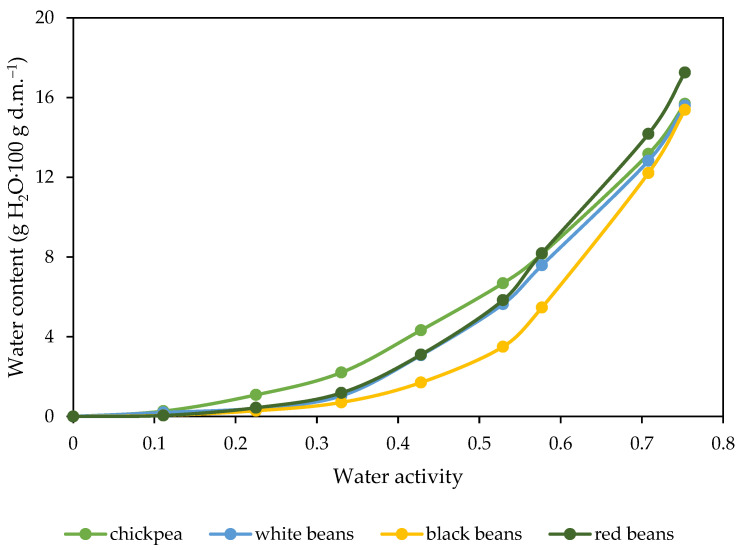
Water vapor adsorption isotherms of crispbread with legume purée.

Water vapor adsorption isotherms characterize the relationship between the moisture content of the crispbread and the relative humidity of the surrounding environment. Isotherms provide important information on the moisture sorption behavior of crispbread, which is essential for its shelf-life stability, texture, and overall quality. Water vapor adsorption isotherms of crispbread were characterized by a characteristic sigmoid shape, typical of products containing a significant amount of proteins and/or carbohydrates [73]. Several studies have observed a similar course of water sorption isotherms of legumes (common beans, chickpeas, lupine and broad beans) [74,75]. The isotherm of crispbread with black bean purée was the lowest in the entire range of water activity and the chickpea purée sample was the highest (Figure 5).

The largest differences between the samples occurred in the water activity range of 0.428 to 0.529, and the equilibrium water content in crispbread based on chickpea purée was approximately 2.2% higher than in crispbread with black bean purée. Differences in the amount of adsorbed water between samples may result from differences in the structure of crispbread, as well as the chemical composition of the purée. The structure of crispbread with chickpea purée was more open, which facilitated the absorption of water vapor from the surrounding environment.

Legumes, compared to some fruits and vegetables, are characterized by lower equilibrium moisture values in the entire range of water activity due to their high content of insoluble compounds [76]. Boucheham et al. [77] showed that powders from legumes, faba bean, and chickpea powders have a higher water adsorption capacity than cereal powders (semolina, rice, and maize) in the entire range of relative humidity (0–95%). The authors pointed to differences in the chemical composition of the powders, including a higher ash content in legume powders compared to cereals, and especially a high protein content. In a complex system, the behavior of each type of compound in water affects the behavior of the entire system [77].

### 2.6. Sensory Evaluation of Crispbread with Legume Purée

**Figure 6:**
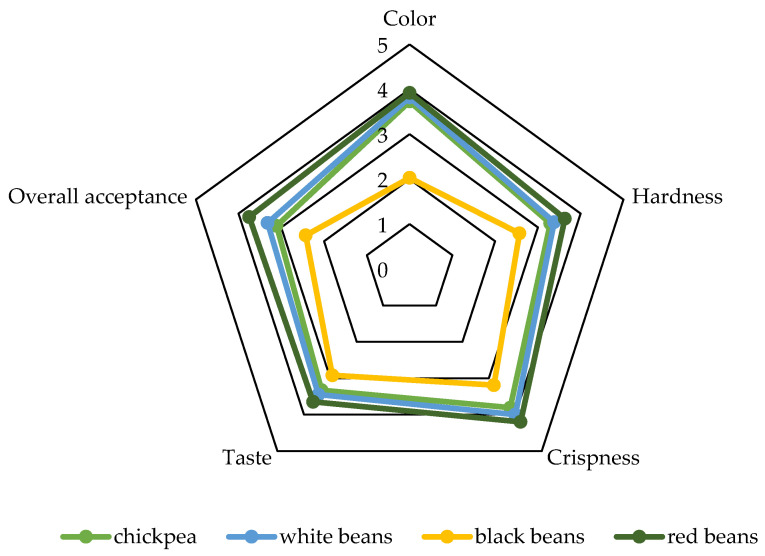
Sensory evaluation of crispbreads with legume purée on a 5-point scale.

Sensory evaluation was used to initially assess the reception of the newly developed crispbread. Color, hardness, crispiness, taste, and overall acceptability were assessed (Figure 6). This was an approach intended only for initial verification. Crispbread with red beans received higher scores compared to other variants. In turn, crispbread with black beans received the lowest scores. It appears that differences in the structure of crispbread with black beans could play an important role in creating a harder texture. In addition, higher values of water content and activity could have softened the crispbread with black beans. The lowest score for crispbread with black beans was for color (2.02).

In turn, the evaluation of the appearance of crispbread without the addition of freeze-dried blackberry powder was similar to that of crispbread with 5% addition [78]. The intensity of taste also determines the overall acceptability of crispbread. For example, in terms of taste (5.64 on a 9-point scale), rice waffles with sea salt were rated the highest among seven crispbread samples [79]. In this study, the taste was rated low, which could have been influenced by the lack of salt added to the recipe. Additionally, organic acids (mainly lactic acid) are responsible for the acidification of the rye dough, which is a key factor in promoting proteolysis, and in turn plays a key role in the production of flavor and volatile organic compounds. Such compounds shape the characteristic flavor and aroma profile and thus affect the overall acceptability of rye crispbread [80]. In turn, the addition of phytosterols to rye crispbread makes it harder and crispier [81].

## 3. Materials and Methods

### 3.1. Materials

Legumes (chickpea, white beans, black beans and red kidney beans) were purchased from the local market. High methoxy citrus pectin (64–67% degree of esterification; JRS Silvateam Ingredients Srl, Bergamo, Italy) was used as a gelling agent and glycerol as a plasticizer. All reagents were of analytical grade and were purchased from Avantor Performance Materials Poland S.A. (Gliwice, Poland).

### 3.2. Legume Solution Preparation

The legumes were soaked in water for 12 h. After the soaking time, the raw materials were separated from the water. The seeds were then poured with water and boiled in a pot for 2 h (the ratio of seeds to water was 1:4 *w*/*w*). The cooked seeds were drained and then left to cool. After cooling, the seeds were ground with a Grindomix GM 200 knife mill (Retsch, Hann, Germany) until a smooth mass was obtained (in the case of white beans and chickpeas, the grinding time was 2 × 20 s, at knife speeds ranging from 3000 to 5000 rpm, while in the case of black and red beans, the grinding process was repeated several times (4 to 6 times) for 30 s each, rotating the knife between 3000 and 5000 rpm until puréed).

Aqueous solution was produced using citrus pectin at a concentration of 5% and glycerol as a plasticizer at 50% relative to pectin (2.5 g per 100 g of water). The solution was heated at 60 °C for 15 min at 250 rpm using an RCT basic IKAMAG magnetic stirrer (IKA Poland, Warsaw, Poland) to obtain a uniform film-forming solution. The solution was then cooled and the legume purée was added at a concentration of 50:50 *w*/*w*.

### 3.3. Legume Crispbread Preparation

The legume solutions were poured onto sheets at a speed of 10 mm/s and a layer thickness of 2500 µm using a Zehntner ZAA 2300 automatic film applicator (Zehntner GmbH Testing Instruments, Sissach, Switzerland), followed by drying at 50 °C for approximately 3 h using the SUP-65 WG laboratory dryer model (WAMED S.A., Warsaw, Poland). Then, all crispbreads were removed from the sheets and packed with the PA/PE 70 T-FLEX 70 polyamide-polyethylene packaging film (Pakmar, Warsaw, Poland) and closed in an air atmosphere using the PP5.4 packaging machine (Tepro S.A, Koszalin, Poland). The samples were stored in the KBF 720 climate chamber model (Binder, Tuttlingen, Germany) at 25 °C and a relative humidity of 30% in an environment without access to light. Before the study of water vapor sorption isotherms and Fourier transform infrared spectroscopy, to reduce the moisture content, the samples were dried at a temperature of 30 °C and a pressure of 1.5 kPa for 48 h using a vacuum dryer (Memmert V0 500, Memmert GmbH, Schwabach, Germany). The dried samples were then kept in a desiccator on phosphorus pentoxide (P_2_O_5_).

### 3.4. Rheological Properties of Legume Solutions

The determination of rheological characteristics was carried out on a Haake MARS 40 rheometer (ThermoFisher Scientific Inc., Erlangen, Germany). The pectin solution with the addition of legume purée was tested at a temperature of 25 °C on a parallel plate (60 mm) measuring system with a shear rate ranging from 0.01 to 100 s^−1^. The apparent viscosity of the solutions was determined for the shear rate 50 s^−1^. The results were compiled using the Rheowin Job Manager (Haake) program. For the mathematical description of the flow curves of solutions, the Ostwald–de Waele model was used (Equation (1)) [82].

(1) $$\tau =k{\left(\dot{\gamma }\right)}^{n}$$

where *τ* is the shear stress (Pa), *γ* is the shear rate (s^−1^), *k* is the consistency coefficient (Pa·s^n^), and *n* is the flow index.

### 3.5. Water Content of Crispbread

The water content was determined in triplicate using the drying method at 105 °C for 4 h with the laboratory dryer model SLW 115 SMART PRO (POL-EKO Aparatura Sp.j., Wodzisław Śląski, Poland) and the analytical weight model AE240 (Mettler Toledo, Warsaw, Poland) with precision ± 0.0001. Then, the % of water content in the sample was calculated.

### 3.6. Water Activity of Crispbread

The water activity of the crispbread was determined after drying with an accuracy of ± 0.003, using an AquaLab series 3 model TE apparatus (Decagon Devices, Pullman, WA, USA) following the manufacturer’s instructions. Measurements were made at 25 °C in triplicate.

### 3.7. Color of Crispbread

Color measurement was performed in 6 repetitions using a Minolta colorimeter (model CR-400, Konica Minolta, Tokyo, Japan) in the CIE L*a*b* system, where L* is the brightness, and a* and b* are the trichromatic coordinates.

### 3.8. Structure of Crispbread

The samples were fixed in a metallic cylindrical layer using PELCO carbon paste with a diameter of 9 mm (Pik Instruments Sp. z o.o., Piaseczno, Poland) and analyzed using the scanning electron microscope model TM3000 (Hitachi High Tech, Tokyo, Japan) at a magnification of 200×. Photographs of the crispbread surface were taken using a VHX-7000 series digital microscope (Keyence, Itasca, IL, USA) at a magnification of 30×.

### 3.9. FT-IR Spectra of Crispbread

Fourier transform infrared (FT-IR) spectra were determined using the total attenuated reflection (ATR) technique with a Cary-630 spectrometer model (Agilent Technologies, Cary, NC, USA). The spectra of the analyzed samples were recorded in the absorption range of 4000–650 cm^–1^ at the resolution of 4 cm^–1^. Each spectrum was an average of 32 interferograms and was presented as a dependence of the absorbance on the wavenumber.

### 3.10. Water Vapor Adsorption Isotherms of Crispbread

The water vapor adsorption isotherms of crispbread were determined using a dynamic vapor sorption analyzer AQUADYNE DVS-2HT (Quantachrome Instruments by Anton Paar, Warsaw, Poland). Crispbread samples 20 ± 1 mg were loaded into a sample glass pan and exposed to a series of relative humidity (RH) (0, 10, 20, 30, 40, 50, 60, and 75%, respectively) until the sample reached equilibrium at 25 °C. The equilibrium criterion at each RH was the percentage rate of mass change with time (dm/dt) < 0.002% min^−1^ in 10 min. The experimental data points were analyzed using Microsoft Excel 2019 together with the aquaWin^TM^ 2.0 analysis software. Data were processed using OriginPro 8.0 software (OriginLab Corporation, Northampton, MA, USA).

### 3.11. Sensory Evaluation of Crispbread

The sensory evaluation of the crispbread was performed using a 5-point rating scale among 30 people. The evaluators were students and employees of the Faculty of Food Technology in the age group of 19–50 years. The quality attributes evaluated were color, hardness, crispness, taste, and overall acceptance of the products.

### 3.12. Statistical Analysis

Statistical analysis was performed using Statistica 10.0 (StatSoft Inc., Tulsa, OK, USA). One-way analysis of variance (ANOVA) was performed with the post hoc Tukey’s test to detect significant differences in the properties of the samples at the significance level used, 0.05.

## 4. Conclusions

The study of vegetable crispbreads containing legume purées presents promising opportunities for the development of functional and nutritious food products. Rheological analysis revealed the influence of legume purées on fluidity and viscosity, highlighting their potential as functional ingredients. Additionally, the characteristics of the crispbread recipes showed low water activity (below 0.19) and a water content (below 3.6%), which extends their shelf life. Structural analysis revealed morphological differences depending on the type of legume purée, providing information on the textural characteristics. FT-IR provided valuable information on the chemical composition, highlighting the distinctive spectral features associated with different legumes. Additionally, the water vapor adsorption isotherms allowed us to explain differences in water sorption capacity, reflecting the interaction of the crispbread structure and composition. Overall, this study opens the way to developing innovative crispbread recipes using legumes. Based on the research conducted, the recipe for crispbread with red bean purée seems to be the best option. These findings provide a promising basis for future research and product development initiatives aimed at expanding the range of nutritious and attractive crispbread offerings on the market.

## Figures and Tables

**Table 1:** Rheological parameters of legume solutions.

Sample	*n*	*k* (Pa·s^n^)	R^2^	*η* (Pa·s)
citrus pectin	0.80 ± 0.01 ^e^	2.18 ± 0.19 ^a^	0.999	1.02 ± 0.11 ^a^
chickpea	0.29 ± 0.02 ^b^	158.63 ± 13.63 ^d^	0.989	9.74 ± 0.19 ^cd^
white beans	0.18 ± 0.01 ^a^	243.37 ± 13.68 ^e^	0.931	10.34 ± 0.91 ^d^
black beans	0.38 ± 0.01 ^c^	92.34 ± 10.62 ^c^	0.997	8.23 ± 0.69 ^c^
red beans	0.42 ± 0.01 ^d^	53.75 ± 10.78 ^b^	0.999	5.53 ± 1.13 ^b^

Values are mean ± standard deviation. Different letters (^a–e^) in the same column represent statistical differences (*p* < 0.05).

**Table 2:** Water content, water activity, and color of crispbread with legume purée.

Crispbread with Legume	Water Content (%)	Water Activity	L*	a*	b*
chickpea 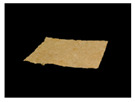	3.58 ± 0.19 ^a^	0.173 ± 0.001 ^b^	71.03 ± 0.86 ^c^	4.80 ± 0.25 ^c^	39.73 ± 0.21 ^d^
white beans 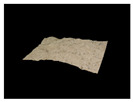	3.42 ± 0.12 ^a^	0.156 ± 0.001 ^a^	74.94 ± 0.43 ^d^	3.05 ± 0.07 ^a^	23.79 ± 0.46 ^c^
black beans 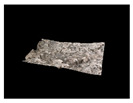	4.12 ± 0.29 ^a^	0.185 ± 0.001 ^c^	34.46 ± 1.24 ^a^	3.84 ± 0.13 ^b^	3.30 ± 0.07 ^a^
red beans 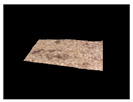	3.16 ± 0.24 ^a^	0.156 ± 0.001 ^a^	47.74 ± 0.93 ^b^	10.05 ± 0.29 ^d^	10.12 ± 0.13 ^b^

Values are mean ± standard deviation. Different letters (^a–d^) in the same column represent statistical differences (*p* < 0.05).

## Data Availability

Data will be available upon reasonable request.

## References

[1] Bolarinwa I.F., Al-Ezzi M.F.A., Carew I.E., Muhammad K. (2019). Nutritional Value of Legumes in Relation to Human Health: A Review. Adv. J. Food Sci. Technol..

[2] Ogrodowczyk A.M., Drabińska N. (2021). Crossroad of tradition and innovation—The application of lactic acid fermentation to increase the nutritional and health-promoting potential of plant-based food products—A review. Polish J. Food Nutr. Sci..

[3] Tas A.A., Shah A.U. (2021). The replacement of cereals by legumes in extruded snack foods: Science, technology and challenges. Trends Food Sci. Technol..

[4] Alcázar-Valle M., Lugo-Cervantes E., Mojica L., Morales-Hernández N., Reyes-Ramírez H., Enríquez-Vara J.N., García-Morales S. (2020). Bioactive compounds, antioxidant activity, and antinutritional content of legumes: A comparison between four Phaseolus species. Molecules.

[5] Pereira A., Ramos F., Sanches Silva A. (2022). Lupin (*Lupinus albus* L.) Seeds: Balancing the Good and the Bad and Addressing Future Challenges. Molecules.

[6] Carbas B., Machado N., Pathania S., Brites C., Rosa E.A.S., Barros A.I.R.N.A. (2023). Potential of Legumes: Nutritional Value, Bioactive Properties, Innovative Food Products, and Application of Eco-friendly Tools for Their Assessment. Food Rev. Int..

[7] Affrifah N.S., Uebersax M.A., Amin S. (2023). Nutritional significance, value-added applications, and consumer perceptions of food legumes: A review. Legum. Sci..

[8] Keskin S.O., Ali T.M., Ahmed J., Shaikh M., Siddiq M., Uebersax M.A. (2022). Physico-chemical and functional properties of legume protein, starch, and dietary fiber—A review. Legum. Sci..

[9] Aluko R.E., Mofolasayo O.A., Watts B.M. (2009). Emulsifying and foaming properties of commercial yellow pea (*Pisum sativum* L.) seed flours. J. Agric. Food Chem..

[10] Karaca A.C., Low N., Nickerson M. (2011). Emulsifying properties of chickpea, faba bean, lentil and pea proteins produced by isoelectric precipitation and salt extraction. Food Res. Int..

[11] Klupšaitė D., Juodeikienė G. (2015). Legume: Composition, protein extraction and functional properties. A review. Chem. Technol..

[12] Vaz Patto M.C., Amarowicz R., Aryee A.N.A., Boye J.I., Chung H.J., Martín-Cabrejas M.A., Domoney C. (2015). Achievements and Challenges in Improving the Nutritional Quality of Food Legumes. CRC Crit. Rev. Plant Sci..

[13] Mefleh M., Pasqualone A., Caponio F., Faccia M. (2022). Legumes as basic ingredients in the production of dairy-free cheese alternatives: A review. J. Sci. Food Agric..

[14] Mulla M.Z., Subramanian P., Dar B.N. (2022). Functionalization of legume proteins using high pressure processing: Effect on technofunctional properties and digestibility of legume proteins. LWT.

[15] Lopes J.S., Simão R.d.S., Mendes G.d.S., Demarco M., de Moraes J.O., Hayashi L., Laurindo J.B., Tribuzi G. (2023). Kappaphycus alvarezii flours as an ingredient for seaweed-enriched, rice-based, snacks: Raw algae pretreatment and physical properties of the dough and snacks. Int. J. Food Sci. Technol..

[16] Mousa M.M.H., El-Magd M.A., Ghamry H.I., Alshahrani M.Y., El-Wakeil N.H.M., Hammad E.M., Asker G.A.H. (2021). Pea peels as a value-added food ingredient for snack crackers and dry soup. Sci. Rep..

[17] Purohit A.S., Reed C., Mohan A. (2016). Development and evaluation of quail breakfast sausage. LWT.

[18] Turfani V., Narducci V., Durazzo A., Galli V., Carcea M. (2017). Technological, nutritional and functional properties of wheat bread enriched with lentil or carob flours. LWT.

[19] Verma A.K., Banerjee R., Sharma B.D. (2015). Quality characteristics of low fat chicken nuggets: Effect of salt substitute blend and pea hull flour. J. Food Sci. Technol..

[20] Foschia M., Horstmann S.W., Arendt E.K., Zannini E. (2017). Legumes as Functional Ingredients in Gluten-Free Bakery and Pasta Products. Annu. Rev. Food Sci. Technol..

[21] Khairuddin M.A.N., Lasekan O. (2021). Gluten-free cereal products and beverages: A review of their health benefits in the last five years. Foods.

[22] Schmidt H.d.O., de Oliveira V.R. (2023). Overview of the Incorporation of Legumes into New Food Options: An Approach on Versatility, Nutritional, Technological, and Sensory Quality. Foods.

[23] Kaur R., Prasad K. (2021). Technological, processing and nutritional aspects of chickpea (*Cicer arietinum*)—A review. Trends Food Sci. Technol..

[24] Yaver E. (2022). Novel crackers incorporated with carob and green lentil flours: Physicochemical, textural, and sensory attributes. J. Food Process. Preserv..

[25] López-Martínez A., Azuara-Pugliese V., Sánchez-Macias A., Sosa-Mendoza G., Dibildox-Alvarado E., Grajales-Lagunes A. (2019). High protein and low-fat chips (snack) made out of a legume mixture. CYTA-J. Food.

[26] Kasapila W., Mwangwela A.M., Njera D., Matumba L., Ng’ong’ola Manani T., Banda R., Nyirenda L. (2024). Sensory acceptability and nutritional quality of composite bread with added purée from biofortified beans in Malawi. Legum. Sci..

[27] Uruakpa F.O., Fleischer A. (2016). Sensory and Nutritional Attributes of Black Bean Brownies. Am. J. Food Sci. Nutr..

[28] Romanchik-Cerpovicz J.E., Jeffords M.J.A., Onyenwoke A.C. (2019). College student acceptance of chocolate bar cookies containing purée of canned green peas as a fat-ingredient substitute. J. Culin. Sci. Technol..

[29] Richardson A.M., Tyuftin A.A., Kilcawley K.N., Gallagher E., O’sullivan M.G., Kerry J.P. (2021). The application of puréed butter beans and a combination of inulin and rebaudioside a for the replacement of fat and sucrose in sponge cake: Sensory and physicochemical analysis. Foods.

[30] Goryńska-Goldmann E. (2017). Bulding competetitive adventage through product innovations based on raw material modyfications. J. Agribus. Rural Dev..

[31] Rachwa-Rosiak D., Nebesny E., Budryn G. (2015). Chickpeas—Composition, Nutritional Value, Health Benefits, Application to Bread and Snacks: A Review. Crit. Rev. Food Sci. Nutr..

[32] Singhal S., Swami Hulle N.R. (2022). Citrus pectins: Structural properties, extraction methods, modifications and applications in food systems—A review. Appl. Food Res..

[33] Nindo C.I., Tang J., Powers J.R., Takhar P.S. (2007). Rheological properties of blueberry purée for processing applications. LWT.

[34] Dankar I., Haddarah A., Sepulcre F., Pujolà M. (2020). Assessing mechanical and rheological properties of potato purée: Effect of different ingredient combinations and cooking methods on the feasibility of 3d printing. Foods.

[35] Picot-Allain M.C.N., Ramasawmy B., Emmambux M.N. (2022). Extraction, Characterisation, and Application of Pectin from Tropical and Sub-Tropical Fruits: A Review. Food Rev. Int..

[36] Mohammed I., Ahmed A.R., Senge B. (2012). Dough rheology and bread quality of wheat-chickpea flour blends. Ind. Crops Prod..

[37] Iagher F., Reicher F., Ganter J.L.M.S. (2002). Structural and rheological properties of polysaccharides from mango (*Mangifera indica* L.) pulp. Int. J. Biol. Macromol..

[38] Sousa A.G., Nielsen H.L., Armagan I., Larsen J., Sørensen S.O. (2015). The impact of rhamnogalacturonan-I side chain monosaccharides on the rheological properties of citrus pectin. Food Hydrocoll..

[39] Liaotrakoon W., de Clercq N., van Hoed V., van de Walle D., Lewille B., Dewettinck K. (2013). Impact of Thermal Treatment on Physicochemical, Antioxidative and Rheological Properties of White-Flesh and Red-Flesh Dragon Fruit (*Hylocereus* spp.) Purées. Food Bioprocess Technol..

[40] Sharma M., Kristo E., Corredig M., Duizer L. (2017). Effect of hydrocolloid type on texture of puréed carrots: Rheological and sensory measures. Food Hydrocoll..

[41] Lin T., Fernández-Fraguas C. (2020). Effect of thermal and high-pressure processing on the thermo-rheological and functional properties of common bean (*Phaseolus vulgaris* L.) flours. LWT.

[42] Klug T.V., Martínez-Sánchez A., Gómez P.A., Collado E., Aguayo E., Artés F., Artés-Hernández F. (2017). Improving quality of an innovative pea purée by high hydrostatic pressure. J. Sci. Food Agric..

[43] Pang Z., Cao J., Li H., Chen C., Liu X. (2020). Rheology and tribology properties of cereal and legume flour paste from different botanical origins. J. Food Sci..

[44] Min B., Lim J., Ko S., Lee K.G., Lee S.H., Lee S. (2011). Environmentally friendly preparation of pectins from agricultural byproducts and their structural/rheological characterization. Bioresour. Technol..

[45] Janowicz M., Kadzińska J., Ciurzyńska A., Szulc K., Galus S., Karwacka M., Nowacka M. (2023). The Structure-Forming Potential of Selected Polysaccharides and Protein Hydrocolloids in Shaping the Properties of Composite Films Using Pumpkin Purée. Appl. Sci..

[46] Kozioł A., Środa-Pomianek K., Górniak A., Wikiera A., Cyprych K., Malik M. (2022). Structural Determination of Pectins by Spectroscopy Methods. Coatings.

[47] Krokida M.K., Maroulis Z.B., Saravacos G.D. (2001). Rheological properties of fluid fruit and vegetable purée products: Compilation of literature data. Int. J. Food Prop..

[48] Konrade D., Klava D., Gramatina I. (2017). Cereal Crispbread Improvement with Dietary Fibre from Apple by-Products. CBU Int. Conf. Proc..

[49] Konrade D., Klava D. (2017). Total Content of Phenolics and Antioxidant Activity in Crispbreads with Plant By-product addition. Rural Sustain. Res..

[50] Leonova S., Badamshina E., Koshchina E., Kalugina O., Gareeva I., Leshchenko N. (2022). Triticale flour in bakery and rusk products. Food Sci. Technol. Int..

[51] Colla K., Gamlath S. (2015). Inulin and maltodextrin can replace fat in baked savoury legume snacks. Int. J. Food Sci. Technol..

[52] Sandulachi E. (2012). Water Activity Concept and Its Role in Food Preservation.

[53] Tapia M.S., Alzamora S.M., Chirife J. (2020). Effects of Water Activity (aw) on Microbial Stability: As a Hurdle in Food Preservation. Water Activity in Foods: Fundamentals and Applications.

[54] Amarowicz R., Pegg R.B. (2008). Legumes as a source of natural antioxidants. Eur. J. Lipid Sci. Technol..

[55] Aguilera Y., Mojica L., Rebollo-Hernanz M., Berhow M., De Mejía E.G., Martín-Cabrejas M.A. (2016). Black bean coats: New source of anthocyanins stabilized by β-cyclodextrin copigmentation in a sport beverage. Food Chem..

[56] Kumar P., Yadav S., Singh M.P. (2020). Possible involvement of xanthophyll cycle pigments in heat tolerance of chickpea (*Cicer arietinum* L.). Physiol. Mol. Biol. Plants.

[57] Monnet A.F., Laleg K., Michon C., Micard V. (2019). Legume enriched cereal products: A generic approach derived from material science to predict their structuring by the process and their final properties. Trends Food Sci. Technol..

[58] Jost T., Henning C., Heymann T., Glomb M.A. (2021). Comprehensive Analyses of Carbohydrates, 1,2-Dicarbonyl Compounds, and Advanced Glycation End Products in Industrial Bread Making. J. Agric. Food Chem..

[59] Anton A.A., Gary Fulcher R., Arntfield S.D. (2009). Physical and nutritional impact of fortification of corn starch-based extruded snacks with common bean (*Phaseolus vulgaris* L.) flour: Effects of bean addition and extrusion cooking. Food Chem..

[60] Aoyagi T., Oshima T., Imaizumi T. (2021). Quantitative characterization of individual starch grain morphology using a particle flow analyzer. LWT.

[61] Wu T., Wang L., Li Y., Qian H., Liu L., Tong L., Zhou X., Wang L., Zhou S. (2019). Effect of milling methods on the properties of rice flour and gluten-free rice bread. LWT.

[62] Cornejo-Ramírez Y.I., Martínez-Cruz O., Del Toro-Sánchez C.L., Wong-Corral F.J., Borboa-Flores J., Cinco-Moroyoqui F.J. (2018). The structural characteristics of starches and their functional properties. CYTA-J. Food.

[63] Santos E.E., Amaro R.C., Bustamante C.C.C., Guerra M.H.A., Soares L.C., Froes R.E.S. (2020). Extraction of pectin from agroindustrial residue with an ecofriendly solvent: Use of FTIR and chemometrics to differentiate pectins according to degree of methyl esterification. Food Hydrocoll..

[64] Jorge A.M.S., Gaspar M.C., Henriques M.H.F., Braga M.E.M. (2023). Edible films produced from agrifood by-products and wastes. Innov. Food Sci. Emerg. Technol..

[65] Van Hung P., Anh M.N.T., Hoa P.N., Phi N.T.L. (2021). Extraction and characterization of high methoxyl pectin from *Citrus maxima* peels using different organic acids. J. Food Meas. Charact..

[66] Rozenberg M., Lansky S., Shoham Y., Shoham G. (2019). Spectroscopic FTIR and NMR study of the interactions of sugars with proteins. Spectrochim. Acta-Part A Mol. Biomol. Spectrosc..

[67] Azeredo H.M.C., Morrugares-Carmona R., Wellner N., Cross K., Bajka B., Waldron K.W. (2016). Development of pectin films with pomegranate juice and citric acid. Food Chem..

[68] Konrade D. (2023). Rheological Properties. One-Two-Dimensional Fluids.

[69] Johnson J.B., Walsh K., Naiker M. (2020). Application of infrared spectroscopy for the prediction of nutritional content and quality assessment of faba bean (*Vicia faba* L.). Legum. Sci..

[70] Van S.J.J.G., Tournois H., Wit D.D., Vliegenthart J.F.G. (1995). Short range structure in partially crystalline potato starch determined with attenuated total reflectance FTIR. Carbohydr. Res..

[71] Ambigaipalan P., Hoover R., Donner E., Liu Q., Jaiswal S., Chibbar R., Nantanga K.K.M., Seetharaman K. (2011). Structure of faba bean, black bean and pinto bean starches at different levels of granule organization and their physicochemical properties. Food Res. Int..

[72] Dome K., Podgorbunskikh E., Bychkov A., Lomovsky O. (2020). Cambios en el grado de cristalinidad del almidón que tiene diferentes tipos de estructura cristalina después pretratamiento mecánico. Polímeros.

[73] Peleg M. (2020). Models of Sigmoid Equilibrium Moisture Sorption Isotherms with and without the Monolayer Hypothesis. Food Eng. Rev..

[74] Menkov N.D. (2000). Moisture sorption isotherms of vetch seeds at four temperatures. J. Agric. Eng. Res..

[75] Pałacha Z., Karwowski W. (2019). Badanie stanu wody w wybranych nasionach roślin strączkowych metodą wykorzystującą izotermy sorpcji. Zesz. Probl. Postępów Nauk Rol..

[76] Moreira R., Chenlo F., Torres M.D. (2009). Simplified algorithm for the prediction of water sorption isotherms of fruits, vegetables and legumes based upon chemical composition. J. Food Eng..

[77] Boucheham N., Galet L., Patry S., Zidoune M.N. (2019). Physicochemical and hydration properties of different cereal and legume gluten-free powders. Food Sci. Nutr..

[78] Różyło R., Wójcik M., Biernacka B., Dziki D. (2019). Gluten-free crispbread with freeze-dried blackberry: Quality and mineral composition. CYTA-J. Food.

[79] Michalak-Majewska M., Muszyński S., Sołowiej B., Radzki W., Gustaw W., Skrzypczak K., Stanikowski P. (2020). Comparative Analysis of Selected Physicochemical and Textural Properties of Bread Substitutes. Acta Univ. Cibiniensis. Ser. E Food Technol..

[80] Ameur H., Tlais A.Z.A., Paganoni C., Cozzi S., Suman M., Di Cagno R., Gobbetti M., Polo A. (2024). Tailor-made fermentation of sourdough reduces the acrylamide content in rye crispbread and improves its sensory and nutritional characteristics. Int. J. Food Microbiol..

[81] Jakubczyk E., Linde M., Gondek E., Kamińska-Dwórznicka A., Samborska K., Antoniuk A. (2015). The effect of phytosterols addition on the textural properties of extruded crisp bread. J. Food Eng..

[82] Mendez D.A., Fabra M.J., Martínez-Abad A., Μartínez-Sanz M., Gorria M., López-Rubio A. (2021). Understanding the different emulsification mechanisms of pectin: Comparison between watermelon rind and two commercial pectin sources. Food Hydrocoll..

